# Immunohistochemical features of giant cell ependymoma of the filum terminale with unusual clinical and radiological presentation

**DOI:** 10.1186/s13000-016-0595-y

**Published:** 2017-01-14

**Authors:** Fernando Candanedo-Gonzalez, Cindy Sharon Ortiz-Arce, Samuel Rosales-Perez, Ana Lilia Remirez-Castellanos, Candelaria Cordova-Uscanga, Armando Gamboa-Dominguez

**Affiliations:** 1 Department of Pathology, Hospital de Oncologia, Centro Medico Nacional Siglo XXI, IMSS, Av Cuauhtemoc #330, Col: Doctores CP, 06720 Mexico City, Mexico; 2 Department of Radiotherapy, Hospital de Oncologia Centro Medico Nacional Siglo XXI, IMSS, Mexico City, Mexico; 3 Department of Radiology, Hospital de Oncologia Centro Medico Nacional Siglo XXI, IMSS, Mexico City, Mexico; 4 Department of Pathology, Instituto Nacional de Ciencias Medicas y Nutricion Salvador Zubiran, Mexico City, Mexico

**Keywords:** Ependymoma, Giant cell, Filum terminale, Immunohistochemistry markers, Atypical clinical presentation

## Abstract

**Background:**

Giant cell ependymoma of the filum terminale is a rare variant, generally manifested as a well-circunscribed intradural mass with an indolent biological behavior.

**Case presentation:**

We describe the case of a 48-year-old Mexican female who non-relevant past medical history, that developed a GCE of the filum terminale. Magnetic resonance imaging and computed tomography revealed the presence of an intra-axial tumor extending from L3 to L5 with extra-medullary invasion. Therefore the tumor was considered unresectable and only incisional biopsy was obtained, establishing the tentative diagnosis of a poorly differentiated neoplasia. A second evaluation of the case revealed the presence of numerous non-cohesive pleomorphic giant cells with intranuclear inclusions and broad eosinophilic cytoplasm, alternating with intermediate size cells with round, hyperchromatic nuclei and forming a perivascular pseudo-rosettes pattern. The ependymal phenotype was supported by light microscopy and corroborated by immunohistochemistry analysis. The patient was subsequently treated with radiotherapy 54Gy. She is alive after a 27-month follow-up, with residual disease, difficulty ambulating and pain.

**Conclusions:**

GCE of filum terminale may have an atypical clinical and radiological presentation, albeit with invasive characteristics and anaplasia on histologic analysis. However, its biological behavior is indolent and associated to longer survival. Due to the presence of giant cells, the differential diagnosis of other primary neoplasias at that site were considered, including paraganglioma, malignant peripheral nerve sheath tumors as well as metastatic malignant melanoma, adrenal carcinoma, thyroid gland carcinoma and urothelial carcinoma, that may all harbor giant cells.

## Background

Ependymomas develop in the neuroaxis from the ependymal cells lining the cerebral ventricles, the central canal of the spinal cord, and the filum terminale. They are rare slow-growing tumors representing 2 to 9% of all neuroepithelial tumors [1]. In adults, 60% of these tumors are found in the spinal cord. The World Health Organization (WHO) has sub-classified ependymomas into cellular, papillary, clear cell, tanycytic, anaplastic, and myxopapillary [1]. Giant cell ependymoma (GCE) is an unusual ependymal tumor sub-type, only recently recognized as diagnostic entity by Zec et al. [2] and it has been included in the WHO classification. This variant is characterized by pleomorphic giant cells intermingled with a typical ependymoma component. These tumors may arise in the supra [3–5], and infratentorial regions [3], the cervical spinal cord [3, 5–8], thoracic spinal cord [3, 9] or involve the filum terminale [2, 5, 10]. Age at presentation ranges between 5 and 89 years [2–10]. In spite of the presence of pleomorphic giant cells, the prognosis has been relatively good in the few reported cases of GCE [2–10]. Due to the rarity of this variant it is difficult to define its clinicopathological and immunohistochemical features. Here, we describe the immunohistochemical features of GCE of filum terminale with high-grade histology, and its evolution over a long follow-up period. Preliminary data were presented at the XVIII International Congress of Neuropathology, Rio de Janeiro, Brazil, 2014 [11] and at the 28^th^ World Congress of Pathology. Cancun, Mexico, 2015 [12].

## Case presentation

**Fig. 1:**
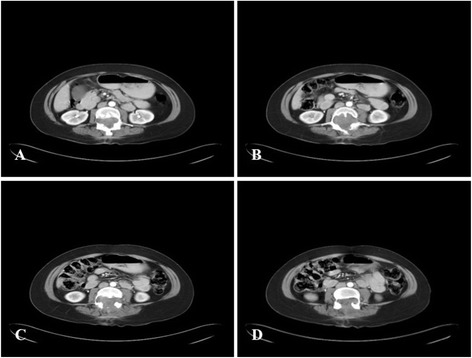
Neuroimaging findings of the GCE from filum terminale. **a-d** Axial gadolinium-enhanced L3-L5-weighted CT image demonstrated an intradural non-encapsulated heterogeneously enhanced solid mass attached to the filum terminale

**Fig. 2:**
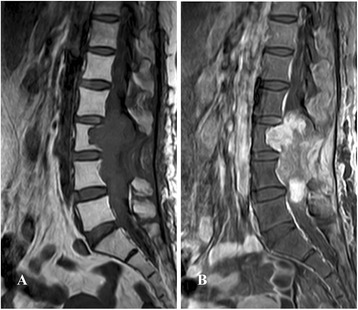
Sagittal MRI showed a large infiltrative mass in the filum terminale. **a** Hyperintensity MRI T2-weighted sequence; and (**b**) Gadolinium-enhanced MRI scan showed a large infiltrative mass with heterogeneous high signals

A previously healthy, 48-years-old mexican woman was admitted at the Hospital de Oncologia, *Centro Medico Nacional Siglo XXI,* with left “pelvic limb” as well as back pain, with loss of strength and balance. Physical examination on admission revealed an ECOG of 1 and difficulty ambulating. Muscle strength of the left lower extremity was 3/5. No adenopathies were found in the head and neck areas, and no tumor was identified in the abdomen. Computed tomography (CT) scan revealed a solid, heterogeneous, non-encapsulated, vascularized intra-axial mass at the L3-L5 lumbar level, measuring 5.0x4.5 cm, that invaded the spinal canal and was fixed to deep planes (Fig. 1a-d). Chest, abdominal and pelvic CT scan revealed no abnormalities. A sagittal magnetic resonance imaging (MRI) of the filum terminale confirmed the presence of a large, expansive and infiltrative spinal mass with associated bone remodeling conditioning image reinforcement. The T2-weighted MRI sequence showed a hyperintense tumor (Fig. 2a), and the gadolinium-enhanced MRI scan revealed a large infiltrative mass with highly heterogeneous signal (Fig. 2b). Also, bilateral compression of the L3-L5 nerve roots was evident. Neurologic examination uncovered no other abnormalities. The imaging differential diagnosis included that of a metastatic tumor. No laboratory abnormalities were reported. Laboratory thyroid function was found T3 89.37 ng/dL (NL 80.00–200.00); T4 9.17 mcg/dL (5.10–12.80); T4L 1.43 ng/dL (NL 0.90–1.70); TSH 0.764 uI/mL (NL 0.270–4.200); TG 9.26 ng/mL (NL 0.10–78.00), vanillylmandelic acid 1.50 ng/mL (NL 1.50–4.8). Serum epinephrine 113 pg/mL (NL 50.00–100.00), serum norepinephrine 596 pg/mL (110.00–658.00), urinary epinephrine of 17.00 μg/day (NL 0.10–20.00), serum dopamine 12.00 pg/mL (NL 10.00–20.00), urinary dopamine of 0.60 μg/day (NL 0.29–1.87 μg/24 h), urinary norepinephrine of 96.00 μg/day (NL 10.00–70.00) and acid 5-hidroxindolacetic of 3.00 mg/24 h. An octreoscan was performed without evidence of involvement other than in the lumbar region. Pre-operatively, the tumor was considered completely unresectable and only incisional biopsy was performed.

### Pathologic findings

Incisional biopsy was obtained for intraoperative evaluation. Macroscopically heterogeneous solid tumor gray-white with areas of congestive appearance of brown-reddish color was observed. Frozen sections revealed a hypercellular tumor with solid growth and encompassing two cell populations. The most striking population consisted of atypical giant cells with irregular hyperchromatic nuclei, with pseudoinclusions and abundant eosinophilic cytoplasm. These cells were intermingled with polygonal intermediate size cells with no atypia, with hyperchromatic nuclei and scant eosinophilic cytoplasm. Due to the observed degree of nuclear atypia and pleomorphism, the intraoperatory diagnosis was reported as consistent with a poorly differentiated neuroendocrine carcinoma, metastatic to the lumbar spine. However, in definitive sections a diagnosis of GCE was rendered.

**Fig. 3:**
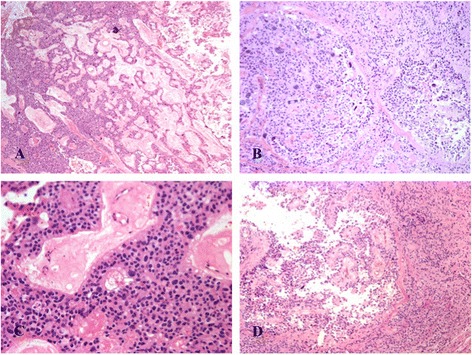
Histological findings of the GCE from filum terminale: **a** At low magnification neoplasia shows trabecular growth pattern and scalloping immersed in a fibrillary stroma (Hematoxylin and eosin, 100X); **b** Neoplasia shows solid growth pattern with formation of nodules surrounded by fibrosis (Hematoxylin and eosin, 100X); **c** Perivascular pseudorosette and ependymal channels lined by monomorphic medium-sized cubic cells (Hematoxylin and eosin 200X); **d** Low power view of pseudo-papillary arrangement in well-differentiated area of this tumor (Hematoxylin and eosin 100X)

**Fig. 4:**
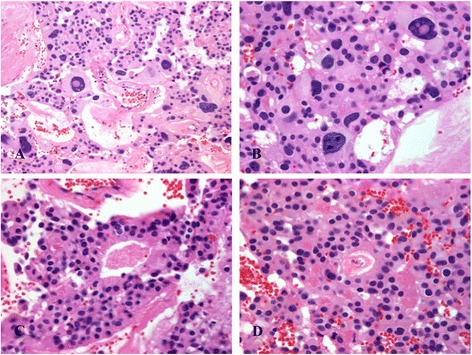
Histological findings of the GCE from filum terminale: **a-b** Pleomorphic giant cells, with abundant eosinophilic cytoplasm, eccentrically located single hyperchromatic large nuclei with prominent nucleoli, and intranuclear cytoplasmic inclusions (Hematoxylin and eosin, 400X); **c-d** Perivascular pseudorosette and ependymal channels lined by monomorphic medium-sized cubic cells (Hematoxylin and eosin 200X)

Microscopically, the tumor showed diffuse, solid, festooned, trabecular, nodular areas with increased cellularity and a focal myxopapillary growth pattern (Fig. 3a-d). At lower power, numerous non-cohesive pleomorphic giant cells with abundant eosinophilic cytoplasm were observed. Nuclei were large, round, hyperchromatic and displayed intranuclear eosinophilic inclusions. They alternated with polygonal medium-sized cells (Fig. 4a-b), forming perivascular pseudorosettes and true rosettes (Fig. 4c-d). No mitoses, microvascular proliferation nor necrosis in pseudopalisading were observed.

### Materials and methods

The tissue was fixed in 10% buffered formaldehyde and paraffin embedded. Hematoxylin and eosin–stained sections were used for diagnosis. For immunohistochemistry (IHC) analysis, 5-μm sections of a representative block were obtained. The following antibodies were used: CD34, c-Kit (CD117), inhibin, S100 protein, CD56, chromogranin, synaptophysin, cytokeratin AE1/AE3, epithelial membrane antigen (EMA), glial fibrillary acidic protein (GFAP), CD99, bcl-2, HMB45, Melan A, PAX5, cytokeratin 20, GATA3, uroplakin III, TTF-1, thyroglobulin, cyclin-D1, p53 and Ki-67, which were performed on an automated immunostainer (Ventana, Biotek System, Tucson, Ariz) with appropriate positive and negative control run concurrently. Briefly, paraffin sections were mounted on charged glass slides, air-dried over-night, and then deparaffinized. To enhance the immunostaining, a heat-induced epitope-retrieval procedure was performed. After incubation with bloking serum, sections were incubated with primary antibodies (Table 1), followed by a biotinylated polyvalent secondary antibody solution. Sections were then incubated with horseradish peroxidase-conjugated avidin-biotin complex, followed by 3,3-diaminobenzidine and hydrogen peroxidase.

**Table 1:** Immunohistochemical antibodies used in this study

Antibody name	Source	Catalog	Dilution	Clone	Staining pattern
		Number			
CD34	BioCare	CM084C	1:200	QBEnd/10	Membranous
c-Kit	BioSB	BSS3221	1:100	4H2	Membranous, cytoplasmic
Inhibin	BioSB	BSB5692	1:50	R1	Cytoplasmic
S100 protein	BioSB	BSB5922	1:100	4C4.9	Cytoplasmic, nuclear
CD56	BioSB	BSB5272	1:100	123C3DS	Cytoplasmic, membranous
Chromogranin	BioSB	BSB5349	1:300	LK2H10	Cytoplasmic
Synaptophysin	BioSB	BSB5950	1:300	Polyclonal	Cytoplasmic
CK AE1/AE3	DAKO	M351501	1:100	No clone	Cytoplasmic
EMA	Biogenex	MU162UC	1:100	Mx5	Cytoplasmic, membranous
GFAP	Zymed	180021	1:100	ZCG-29	Cytoplasmic
CD99	BioSB	BSB5314	1:50	CD99/B5	Cytoplasmic, membranous
bcl-2	Cell Marque	226R-16	1:50	E17	Cytoplasmic
HMB45	DAKO	M0634	1:100	HMB45	Cytoplasmic
Melan A	Biocare	CM125C	1:100	Melan-A A103	Cytoplasmic
PAX5	BioSB	BSP5866	1:100	RBT-Pax-5	Nuclear
Uroplakin III	Cell Marque	3454-16	1:50	AU-1	Cytoplasmic, membranous
Cytokeratin 20	Biocare	CM062C	1:100	Ks20.8	Cytoplasmic
GATA3	BioSB	BSB2674	1:100	LSO-823	Nuclear
TTF-1	Zymed	18-0221	1:100	867G3/1	Nuclear
Thyroglobulin	BioGenex	MUD332UC	1:200	92H11	Cytoplasmic
Cyclin-D1	Biocare	CRM307CK	1:100	SP4	Nuclear
p53	BioGenex	ML236UC	1:500	DX7	Nuclear
Ki-67	Cell Marque	275R-16	1:150	SP6	Nuclear

*c-kit* CD117, *CK* Cytokeratin, *EMA* epithelial membrane antigen, *GFAP* glial fibrillary acidic protein

## Results

### Immunohistochemistry findings

**Fig. 5:**
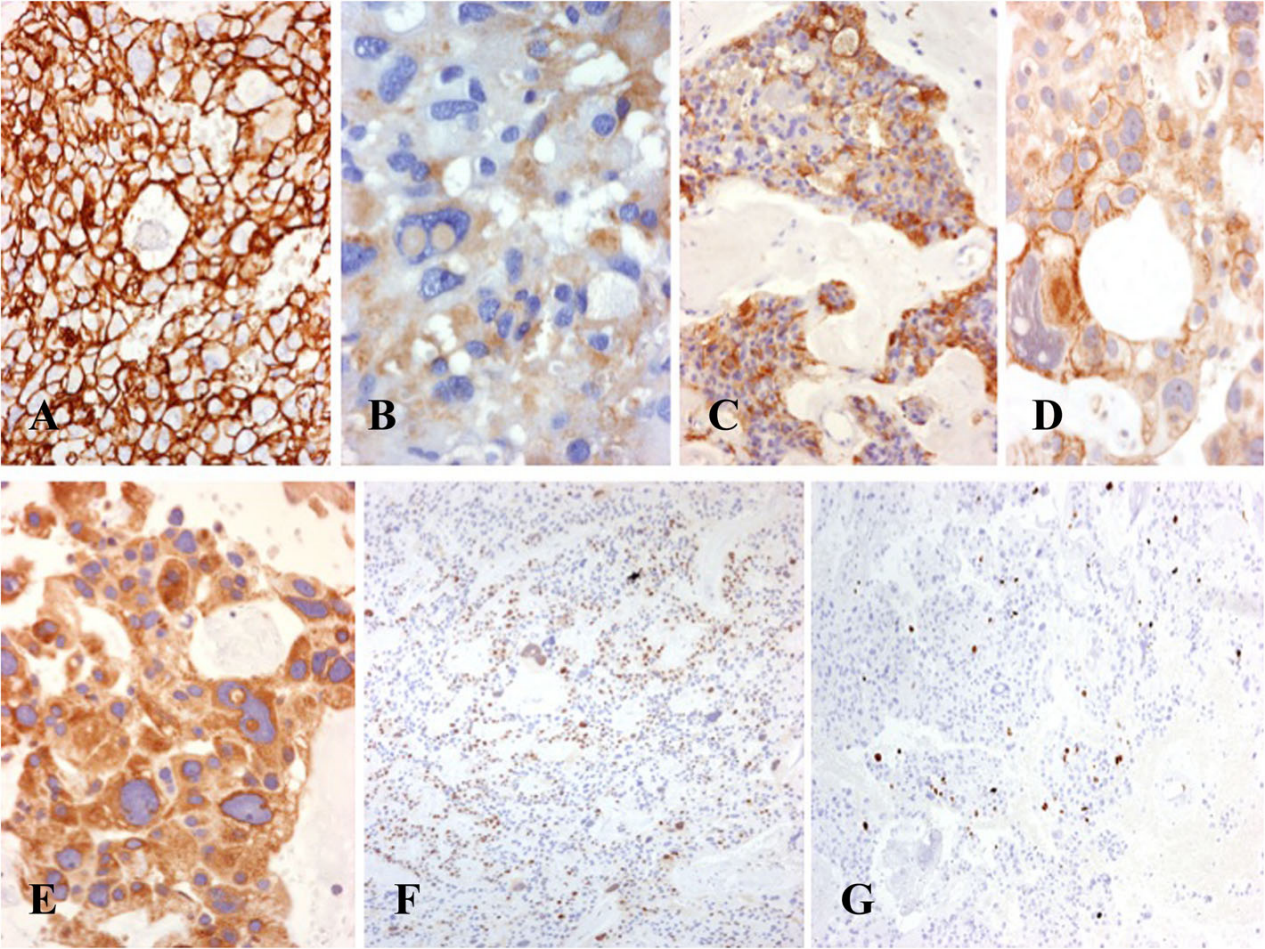
Immunohistochemical findings of the GCE from filum terminale. **a** All neoplastic cells show positivity for CD56, including giant cells; **b** Both giant and medium-sized neoplastic cells show heterogeneous positivity for GFAP; **c** Neoplastic cells show intense positivity for S-100 protein; **d** Neoplastic cells also shows cytoplasmic positivity for CD99; **e** Both giant and medium-sized neoplastic cells shows positivity for bcl-2; **f** Further medium-sized neoplastic cells shows nuclear positivivity for cyclin D1; **g** Neoplastic cells shows a cell proliferation index of 15%

Giant cells and pseudorosette-forming cells were CD56 (Fig. 5a) and GFAP positive (Fig. 5b). They also stained multifocally for S-100 protein (Fig. 5c). A diffuse strong membranous and cytoplasmic dot-like pattern expression of CD99 was found (Fig. 5d). Both giant and small neoplastic cells show positivity for bcl-2 (Fig. 5e). Also, the small neoplastic cells showed nuclear positivity for cyclin D1 and p53 (Fig. 5f). All other markers were negative. The cell proliferation index analyzed by Ki-67 was 15% in the perivascular pseudo-rosette areas and negative in the giant cells (Fig. 5g).

The patient was subsequently treated with spinal column radiotherapy, 54 Gy divided in 28 fractions decreased her symptoms. Twenty-seven months after treatment, the patient is alive, with residual diseases, difficulty ambulating and pain.

## Discussion

Ependymomas of the filum terminale represent 1 to 2% of all spinal tumors, but the GCE is an extremely rare variant [1]. Other than our reported case, 26 cases of GCE have been described in the English literature [2–10]. However, this is the fifth case report of GCE originating in the filum terminale [2, 5, 10]. Unlike the cases reported by Zec et al. [2] ours had an unusual clinical and radiological presentation with a non-encapsulated tumor infiltrating extramedullary portion with soft tissue infiltration. Table 2 summarizes some characteristic of the informed cases of GCE of the filum terminale. We also conducted a review of literature in search of studies on GCE, focusing on the IHC characteristics, but due to the rarity of this variant we found no studies analyzing immunohistochemical markers. In order to identify markers that could potentially be useful in the differential diagnosis with other neoplasm with giant cells. This is the first study that includes of the expression of CD56, CD99, bcl-2 and cyclin D1 in addition to the GFAP and S100 protein.

**Table 2:** Clinicopathological features of currently reported cases of GCE of filum terminale

Reference	Gender	Age	Clinical features	Lesion features
1. Zec et al. (1996) [2]	M	14		Well circumscribed lumbar intradural mass at the L4-L5 level.
2. Zec et al. (1996) [2]	M	14		Well circumscribed enhanced lumbar mass at the L2-L3 level.
3. Shintaku et al. (2009) [10]	F	55	Sensation of heaviness around the waist and the lower limbs three months earlier. After 2 months, lumbago.	Well circumscribed intradural tumor.
4. Li et al. (2012) [5]	F	34	History of tingling and numbness in the right side of body and weakness.	Cystic tumor.
5. Present case (2016)	F	48	Pain in the Left pelvic limb and back pain with loss of strenght and balance.	Infiltrative mass at the L3-L5.

Clinical and radiological aspect of GCE of the filum terminale is nonspecific, consisting of back pain with motor-sensory deficit and may lead to a cauda equina syndrome. MRI, it is the diagnostic test of choice because it allows knowing the extent of the tumor, its relation to central structures and nerve roots and it permits the evaluation of the dissemination in subarachnoid space. MRI is typically isointense in relation to the spinal cord on T1 and hyperintense on T2 sequences. Gadolinium-enhanced MRI scans more frequently reveals ependymomas, which are homogeneously (75% of cases) and tend to be well encapsulated or heterogeneously (25%) enhanced by the contrast agent at a moderate or high intensity, which are not encapsulated [13]. Thus, treatment is surgical with complete resection of the tumors, yielding excellent outcome [2]. However, in larger volume tumors, the pedicles or the posterior vertebral body surface may be eroded. Once the tumor breaks the capsule, it can infiltrate the nerve roots, and this is associated with a high recurrence rate and postsurgical neurologic deficit. Therefore, surgical treatment depends primarily on the size of the tumor and is encapsulation. From an imaging viewpoint, in all previously reported GCE cases, an intramedullary encapsulated mass has been observed in the filum terminale with expansion of the cord [2, 5, 10]. In our case, Gadolinium-enhanced MRI scan revealed a heterogeneously tumor measuring 5 cm in its greatest axis, non-encapsulated tumor and infiltrating and adhering to adjacent nerve tissue. Unfortunately, the tumor could not be completely resected and only a biopsy was obtained for histopathological diagnosis. The patient was only treated with palliative radiotherapy for local disease control.

In general, there is no problem to make the diagnosis of conventional ependymoma in morphological basis. However, GCE is particularly difficult to recognize, especially in the filum terminale, as in our case. Zec et al. [2] have proposed that the cellular pleomorphism presenting GCE is a result of degenerative changes. In our case, the presence of giant pleomorphic cells could have led to confusion with an anaplastic ependymoma. However, we observed no mitotic activity, microvascular proliferation or necrosis with pseudopalisading pattern. Further, we considered that finding giant cells with pseudoinclusions intraoperatively study may be a distractor due to the degree of pleomorphism and nuclear atypia that may lead to the incorrect diagnosis of a high grade malignant tumor; it may also delay a correct diagnosis, if not carefully observed adjacent areas, which are rosettes and pseudorosettes that establish the diagnosis of GCE. It is advisable to include the entire tissue to view the classic ependymoma areas. The diagnosis of GCE is therefore, still, a diagnosis of exclusion. The differential diagnosis of GCE should initially include other intradural extramedullary tumors, particularly those located in the lumbar region, including some benign or malignant, primary or metastatic, tumors both primary and metastatic, but histologically characterized by the presence of giant cells. The differential diagnosis encompasses paragangliomas [14], malignant peripheral nerve sheath tumors (MPNST) [15, 16], and metastatic tumors [17, 18]. The approach to the differential diagnosis should consider the clinical findings, imaging, morphologic and IHC features.

Immunohistochemically, ependymomas are characterized by a diffuse expression of GFAP and S-100 protein and can also express epithelial markers such as EMA [19]. In our case, neoplastic cells expressed both GFAP and S100 protein, but were negative for EMA. We believe the EMA was negative due to its low sensitivity according to other studies that have observed a lower sensitivity of the 72% [20]. Our aim was to test other IHC markers that have been studied in non-giant cell ependymomas and that could possibly be extrapolated to our case. Based in a study published by Lamzabi et al. [21] in myxopapillary ependymomas that expressed CD99 in all cases and CD56 was diffusely positive in 88% cases, we attempted the technique. Other IHC studies have analyzed the expression of bcl-2, p53 and cyclin D1 in ependymomas and they appear to act as prognostic predictors although results are discordant [22]. To date, none of these markers has been tested in GCE. Therefore, we studied the expression of CD99, CD56, bcl-2, p53, and cyclin D1 to further characterize our case, which were positive. We believe that these markers could be useful in the differential diagnosis of these tumors. Since these markers have not been found to co-express together in other tumors with giant cells such as paragangliomas, MPNST, malignant melanomas, adrenal carcinoma, thyroid gland carcinoma or urothelial carcinoma giant cell variant. Table 3 summarizes the immunohistochemical characteristics of GCE and its differential diagnosis with other tumors that may have giant cells.

**Table 3:** Differential diagnosis by immunohistochemistry of GCE with other tumors that may have giant cells

Marker	GCE	Paraganglioma	MPNST	Metastatic melanoma	Adrenal carcinoma	Urothelial carcinoma
S-100 protein	+	+	+/-	+/-	-	-
GFAP	+	-	-	-	-	-
CD56	+	+	-	-	-	-
CD99	+	-	-	-	-	-
Bcl-2	+	-	-	-	-	-
Cyclin D1	+	-	-	-	-	-
Inhibin	-	-	-	-	+	-
HMB45	-	-	-	+	-	-
Melan A	-	-	-	+	-	-
Chromogranin	-	+	-	-	-	-
Synaptophysin	-	+	-	-	-	-
CKAE1/AE3	-	-	-	-	+	-
EMA	+/-	-	-	-	+	-
CK20	-	-	-	-	-	+
Uroplakin III	-	-	-	-	-	+
GATA3	-	-	-	-	-	+
PAX5	-	-	-	-	-	+

*CK* cytokeratin, *EMA* epithelial membrane antigen,*GFAP* Glial fibrillary acidic protein

In this location, one of the differential diagnoses in imaging studies is the paraganglioma of the filum terminale. They occur in adults between the fourth and sixth decades of life and are associated to catecholamine increases that may lead to hypertension [14]. Histologically, are characterized by nesting of chief cells with round or oval nuclei with salt and pepper chromatin pattern; however, occasionally they may harbor giant cells with pseudoinclusions and formation of pseudorosettes. But, nesting cells are more epithelioid-like and by IHC, chief cells are immunoreactive for chromogranin, synaptophysin and CD56, but are CD99 negative [14]. Sustentacular cells may also be identified, which are positive for S-100 protein. Although in our case S-100 protein was expressed in the neoplastic cells, both small cells and giant cells, we did not detect chromogranin or synaptophysin expression. These findings coupled with normal serological and urinary levels of catecholamines in our patient excluded the possibility of a paraganglioma.

Another differential diagnosis to consider by imaging studies is the MPNST. Although, MPNST of the lumbar area are exceptionally rare, they usually develop in the spinal nerve roots and lead to secondary bony changes [15, 16]. MRI of the lumbosacral spine shows a large mass originating in the cauda equina with surrounding bony destruction. These are very aggressive tumors that recur and metastasize with poor survival [15, 16]. In our case, the tumor invaded the nerve roots. However, our patient is currently alive and with no metastatic activity. By IHC both tumors may express S100 protein and PAGF [15, 16]. Therefore, it is convenient to use an antibody panel, which must include CD56, CD99, bcl-2 and cyclin D1, which in our case were positive while in the MPNST are negative.

Metastatic tumors of the intramedullary spinal cord are rare and cause diagnostic and therapeutic problems. Since GCE may have pleomorphic giant cells with pseudoinclusions, we also considered malignant melanoma, adrenal carcinoma, thyroid gland carcinoma, and urothelial carcinoma, among our differential diagnoses. The morphologic distinction between a GCE and metastatic malignant melanoma may be complex. Malignant melanoma metastases to the intramedullary spinal cord are extremely rare and usually show multiple lesions [17]. By IHC, neoplastic cells was HMB45 and Melan-A negative, therefore that possibility was excluded. On the other hand, adrenal carcinomas can metastasize anywhere, although metastases to the spinal cord are also extremely rare [17]. Histologically, they may have neoplastic cells with giant nuclei and extensive necrosis. In our patient the CT showed no evidence of tumors in either adrenal gland. By IHC, neoplastic cells were negative for inhibin. Therefore, this possibility was also excluded. In the preliminary analysis, papillary thyroid carcinoma was also considered due to the presence of a focal papillary pattern and cells with nuclear pseudoinclusions and oxyphilic cytoplasm [23]. But, serum thyroid function tests were within normal parameters and by IHC the neoplastic cells were negative for thyroglobulin and TTF-1, which this possibility was excluded. Finally, we also considered a high-grade urothelial carcinoma variant of giant cells that had metastasized to the spinal cord. Uroplakin III, GATA3, cytokeratin 20, and PAX5 haven been reported to be a useful marker in the identification of an urothelial origin since it is expressed in neoplastic bladder cells [24, 25]. All these markers were negative in our patient, so this neoplasia was also excluded.

## Conclusions

In conclusion, we report a case of GCE of the filum terminale characterized by the presence of giant cells with pleomorphic nuclei and nuclear pseudoinclusions with slow-growing and an indolente clinical behavior. The presence of perivascular pseudo-rosettes and ependymal rossettes is a key histologic feature to confirm the diagnosis of ependymoma. However, due to the presence of giant cells, it is first necessary to make differential diagnosis with other entities. In our case the ependymal origin was suspected in hematoxylin and eosin staining and confirmed by IHC. In this histological variant is convenient to use an antibody panel, which must include GFAP, S-100 protein, EMA, CD56, CD99, bcl-2. An ependymoma with heterogeneously enhancement on MRI suggests the presence of an infiltrating non-encapsulated tumor.

## Abbreviations

CT: Computed tomography
EMA: Epithelial membrane antigen
GCE: Giant cell ependymoma
GFAP: Glial fibrillary acidic protein
Gy: Gray (symbol: Gy) is a derived unit of ionizing radiation dose in the International System of Units
IHC: Immunohistochemistry
MPNST: Malignant peripheral nerve sheath tumors
MRI: Magnetic resonance imaging
WHO: World Health Organization
